# Iodine Supplementation in Pregnancy in an Iodine-Deficient Region: A Cross-Sectional Survey

**DOI:** 10.3390/nu14071393

**Published:** 2022-03-27

**Authors:** Carla A. Lopes, Susana Prazeres, José Martinez-de-Oliveira, Edward Limbert, Manuel C. Lemos

**Affiliations:** 1 CICS-UBI, Health Sciences Research Centre, University of Beira Interior, 6200-506 Covilhã, Portugal; ccasantos5@gmail.com (C.A.L.); jmo@fcsaude.ubi.pt (J.M.-d.-O.); 2 Departamento da Saúde da Criança e da Mulher, Centro Hospitalar Universitário Cova da Beira, 6200-251 Covilhã, Portugal; 3 Laboratório de Endocrinologia, Serviço de Patologia Clínica, Instituto Português de Oncologia de Lisboa Francisco Gentil, 1099-023 Lisboa, Portugal; sprazeres@ipolisboa.min-saude.pt; 4 Serviço de Endocrinologia, Instituto Português de Oncologia de Lisboa Francisco Gentil, 1099-023 Lisboa, Portugal; edwardlimbert@gmail.com

**Keywords:** iodine deficiency, thyroid hormones, pregnancy, supplementation

## Abstract

Iodine deficiency is a common problem in pregnant women and may have implications for maternal and child health. Iodine supplementation during pregnancy has been recommended by several scientific societies. We undertook a cross-sectional survey to assess the efficacy of these recommendations in a European iodine-deficient region. Urinary iodine concentrations (UIC) were determined in pregnant women before (*n* = 203) and after (*n* = 136) the implementation of guidelines for iodine supplementation in pregnancy. Iodine supplementation (200 μg/day) reduced the proportion of pregnant women with severe iodine deficiency (37.4% to 18.0%, *p* = 0.0002). The median UIC increased from 67.6 µg/L to 106.8 µg/L but remained below the recommended target level (>150 µg/L) for pregnant women. In conclusion, iodine supplementation in pregnant women improved iodine status in this iodine-deficient region but was insufficient to achieve recommended iodine levels in pregnancy. Additional measures, such as the adjustment of the dose or timing of supplementation, or universal salt iodization, may be needed.

## 1. Introduction

Pregnancy requires a healthy diet that includes an adequate supply of energy, protein, vitamins, and minerals to meet the increased needs of the mother and the fetus [1]. Poor dietary intake of key micronutrients has been linked to compromised pregnancy outcomes and neonatal health and is a global public health concern [2]. Although adequate food intake remains the preferred means for meeting dietary needs, some micronutrient requirements in pregnancy are difficult to meet with diet alone. In response, some countries have implemented programs for the fortification of selected foods and/or recommendations for the use of dietary supplements [3].

Iodine is an essential element for the synthesis of thyroid hormones and its deficiency remains a public health problem in many regions around the world [4]. Dietary iodine requirements are higher during pregnancy due to increased thyroid hormone production, increased renal iodine excretion, and fetal iodine requirements. Thus, pregnant women are particularly vulnerable to iodine deficiency [5].

Adequate nutritional iodine intake during pregnancy and early childhood is critically important for brain development and maturation. Severe maternal iodine deficiency has been associated with cretinism or impaired neurodevelopment in children as well as obstetric complications [6]. Universal salt iodization or iodine supplementation programs have been shown to prevent these effects in severely iodine-deficient areas [7]. Mild to moderate iodine deficiencies have also been associated with poorer cognitive outcomes in children, although the evidence for this is less robust [8,9].

The median urinary iodine concentration (UIC) is the most frequently used biomarker to assess the iodine status of a population [10]. A median UIC < 100 µg/L in the general population, or <150 µg/L in pregnant women, indicates iodine deficiency [11]. In many parts of the world, including Europe and North America, significant iodine deficiency has been observed in pregnant women [12,13]. Several scientific societies, including the European Thyroid Association [14], the American Thyroid Association [15], and the Endocrine Society [16], currently recommend iodine supplementation for women who are pregnant, breastfeeding, or planning pregnancy, with formulas containing at least 150 μg of iodine/day.

A countrywide cross-sectional survey of pregnant Portuguese women reported in 2010 [17] showed widespread iodine deficiency in this population (median UIC 82.5 μg/L). Consequently, in 2013, the Portuguese health authorities issued recommendations for iodine supplementation (150 to 200 μg/day) in all women during the preconception, pregnancy, and breastfeeding periods [18]. The aim of this study is to assess the impact of these measures on the iodine status of pregnant Portuguese women living in an iodine-deficient region.

## 2. Materials and Methods

### 2.1. Subjects

The study was carried out as a cross-sectional survey of pregnant women attending a hospital-based outpatient clinic (Centro Hospitalar Universitário Cova da Beira, Covilhã, Portugal) for routine pregnancy checkups and screening tests. This regional hospital is located in the hinterland of continental Portugal, which is historically known as an iodine-deficient region [19]. A previous countrywide study [17] showed that pregnant women in this region were among the most severely affected by iodine deficiency. We used the data of 203 pregnant women who had been enrolled for a previous study [17], from June 2006 to March 2007 (mean gestational age ± standard deviation (SD) = 29.0 ± 9.9 weeks). None of these women had taken iodine supplements during pregnancy at the currently recommended levels, as national guidelines to promote iodine supplementation during pregnancy were only established in 2013 [18]. To evaluate the effect of these guidelines on iodine status during pregnancy, we assessed a random sample of 136 pregnant women, from October 2018 to May 2019 (mean gestational age ± SD = 27.8 ± 8.7 weeks). Women with known thyroid disorders were excluded from the study. Women were considered to be receiving iodine supplements if they were taking a daily tablet containing at least 150 µg of iodine, as recommended in national guidelines [18]. The study was approved by the Institutional Ethics Committee of the Centro Hospitalar Universitário Cova da Beira, Covilhã (Ref: 35/2017) and informed consent was obtained from all subjects.

### 2.2. Assessment of Urinary Iodine Concentration

Iodine status was assessed by determining the UIC in morning spot urine samples using a fast colorimetric method appropriate for population studies [20]. Samples from the 2018/19 group of pregnant women were measured by the same method, laboratory, and technician, as those that had been measured in the 2006/07 survey [17]. UIC (µg/L) were assigned to categories of an ordinal variable, as previously described [17].

### 2.3. Statistical Analyses

Median UIC values were calculated by distributing sample concentrations evenly within each category of ordinal variables [17]. The distribution of UIC was compared between the groups of pregnant women using a chi-square test. The proportions of women with severe iodine deficiency (<50 µg/L) and iodine sufficiency (>150 µg/L) were compared between the groups using a one-sided Fisher’s exact test. A *p*-value < 0.05 was considered statistically significant. All analyses were performed using GraphPad Prism (Version 7.04/2017 for Windows, GraphPad Software, San Diego, CA, USA).

## 3. Results

In the 2018/19 group of 136 pregnant women, 111 (81.6%) were taking one of several commercially available tablets containing potassium iodide (providing a daily dose of 200 µg of iodine) since their first prenatal medical visit (GestaCare^®^, Lifewell, Paço d’Arcos, Portugal, https://lifewell.pt/gestacare-gravidez/, accessed 5 March 2022; Matervita^®^, Laboratórios EFFIK, Algés, Portugal, https://www.effik.pt/detalhe_produto.php?cd_produto=70, accessed 5 March 2022; Natalben Supra^®^, Italfarmaco, Algés, Portugal, https://italfarmaco.wixsite.com/italfarmaco2019/p/natalben-supra; or Yodafar^®^, Bial, Coronado, Portugal, https://www.bial.com/pt/produtos/, accessed 5 March 2022). The remaining 25 (18.4%) women were not taking any supplements or were taking multivitamin/mineral tablets containing less than the recommended (150 µg) daily dose of iodine. These 25 women were at an earlier stage of pregnancy than those who were taking iodine supplements (mean gestational age 23.7 vs. 28.7 weeks, *t*-test *p* = 0.009).

**Figure 1:**
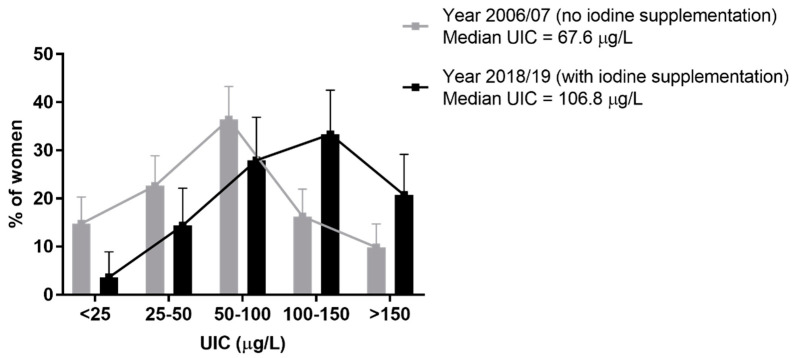
Distribution of urinary iodine concentration (UIC) in untreated (*n* = 203) and iodine-supplemented (*n* = 111) pregnant women, studied in 2006/07 and 2018/19, respectively. Supplementation with a daily dose of 200 µg iodine resulted in a shift towards a higher UIC in pregnancy (chi-square test *p* < 0.0001). For clarity, UIC higher than 150 µg/L have been grouped together. Bars represent percentages (%) of total and error bars represent 95% confidence intervals.

The median UIC in the 2006/07 non-supplemented, 2018/19 non-supplemented, and 2018/19 iodine-supplemented groups were 67.6 µg/L, 50.0 µg/L, and 106.8 µg/L, respectively. The percentage of women with severe iodine deficiency (<50 µg/L) was significantly lower in the 2018/19 iodine-supplemented group, compared with the 2006/07 non-supplemented (18.0% vs. 37.4%, *p* = 0.0002) and with the 2018/19 non-supplemented (18.0% vs. 52.0%, *p* = 0.0008) groups (Table 1). The percentage of women with adequate iodine levels (>150 µg/L) was significantly higher in the 2018/19 iodine-supplemented group, compared with the 2006/07 non-supplemented (20.7% vs. 9.9%, *p* = 0.0069) and with the 2018/19 non-supplemented (20.7% vs. 0%, *p* = 0.0058) groups (Table 1). Overall, the comparison between pregnant women tested in 2006/07, before the launch of national guidelines on iodine supplementation, and pregnant women undergoing supplementation in 2018/19, showed a shift towards a higher UIC in the latter group (*p* < 0.0001) (Table 1 and Figure 1).

The median UIC was lower in later stages of pregnancy, in both the 2006/07 and 2018/19 surveys, although this was not statistically significant (Table 2).

## 4. Discussion

This cross-sectional survey of pregnant women in a European iodine-deficient region showed an improvement in iodine status after national health authorities issued recommendations for iodine supplementation of all women during the preconception, pregnancy, and breastfeeding periods [18]. At the time of this survey, over 80% of pregnant women were taking iodine-supplement tablets (200 µg/day). Compared with pregnant women surveyed before the introduction of iodine supplementation, the proportion of pregnant women with severe iodine deficiency (UIC < 50 µg/L) was reduced to half (37.4% to 18.0%), and those with adequate iodine levels (UIC > 150 µg/L) increased two-fold (9.9% to 20.7%). The median UIC increased from 67.6 µg/L to 106.8 µg/L. However, this increase in median UIC was still insufficient to meet the World Health Organization (WHO) recommended levels for pregnant women (median UIC of at least 150 µg/L) [11].

To achieve an adequate iodine status during pregnancy, the WHO currently recommends a dietary intake of 250 µg of iodine daily, which in iodine-deficient regions can be provided through universal salt iodization or administration of iodine supplements [11]. The widespread problem of iodine deficiency has led medical societies in Europe and North America to recommend that all pregnant women take a daily tablet containing at least 150 µg of iodine [14,15,16]. Health authorities in several countries, including Portugal, have introduced these recommendations into routine pregnancy care [18,21]. However, our data suggest that current recommendations for iodine supplementation during pregnancy may still be insufficient to achieve an adequate iodine status in some iodine-deficient regions.

In our study, the daily dose of iodine supplementation in pregnant women was 200 µg, as this is the content of the most widely available oral tablets containing potassium iodide in Portugal. This dose of supplementation, which is higher than the minimum recommended dose (150 µg), failed to increase the median UIC to recommended levels in pregnancy. This suggests that iodine dietary intake in this iodine-deficient region is at such a low level that it cannot be compensated by currently recommended doses of iodine supplementation. Indeed, a previous survey of pregnant women in this region showed that the median UIC (67.6 µg/L) was among the lowest in continental Portugal prior to iodine supplement implementation [17]. In addition, a study of schoolchildren in this region showed widespread iodine deficiency (median UIC 97.2 µg/L) [19].

It should be noted that iodine supplementation is recommended to be initiated ideally before conception [18], but in our group of pregnant women, supplementation occurred only after women knew they were pregnant. This means that women were likely to already have largely depleted thyroid iodine stores when entering pregnancy and that most of the supplemented iodine may have been taken up by the thyroid for both production of thyroid hormones and rebuilding iodine stores, resulting in a lower iodine excretion in the urine. It remains to be determined whether the results would have been different if women had initiated supplementation before becoming pregnant. As it is unrealistic to expect all pregnancies to be planned for and preceded by a period of iodine supplementation, additional measures to increase the baseline iodine intake in this region, such as universal salt iodization, may be justified. Mandatory salt iodization has already been introduced in at least 124 countries, but not in Portugal, as a strategy to improve the populational iodine intake [22]. This approach could help ensure that all women of reproductive age have higher levels of iodine intake at the start of pregnancy and during the initial stages of fetal development, even before iodine tablets are prescribed. The importance of having adequate iodine stores before pregnancy is illustrated by a study in the United Kingdom that demonstrated a positive association between preconception iodine status and subsequent cognitive function in childhood [23].

In other populations, iodine supplementation has not always fully corrected iodine deficiency in pregnancy [24]. This may be due to the severity of baseline iodine deficiency in these populations and illustrates the importance of monitoring the iodine status of pregnant women even when iodine supplements are routinely prescribed [25].

Our results should be viewed with caution. First, although our measurements of UIC followed WHO recommendations for population surveys [11], we did not measure urinary creatinine to account for urine dilution or other indicators of iodine intake, such as maternal thyroid-stimulating hormone (TSH) and thyroglobulin levels. Second, individual data on iodine supplementation were based on self-reporting and prescription records, but the extent of treatment compliance could not be determined. Third, iodine supplementation was initiated only after conception, and it remains to be elucidated if supplementation before pregnancy would have corrected the iodine deficiency. Last, the use of larger doses of iodine supplements to improve UIC during pregnancy would need careful consideration to avoid the risk of overdosing and causing any harmful effects on the mother or fetus.

In conclusion, our study suggests that, in some iodine-deficient regions, supplementation of pregnant women with currently recommended doses of iodine may be insufficient to achieve an adequate iodine intake and additional measures, such as adjustment of the dose or timing of supplementation, or universal salt iodization, may be needed.

## Figures and Tables

**Table 1:** Urinary iodine concentration (UIC) before (2006/07) and after (2018/19) the launch of national guidelines to promote iodine supplementation during pregnancy.

	Year 2006/07 (No Iodine Supplementation) (*n* = 203; Median UIC = 67.6 µg/L)	Year 2018/19 (No Iodine Supplementation) (*n* = 25; Median UIC = 50.0 µg/L)	Year 2018/19 (200 µg/Day Iodine Supplementation) (*n* = 111; Median UIC = 106.8 µg/L)
UIC (µg/L)	*n* (%)	*n* (%)	*n* (%)
<25	30 (14.8)	5 (20.0)	4 (3.6)
25–50	46 (22.7)	8 (32.0)	16 (14.4)
50–100	74 (36.5)	4 (16.0)	31 (27.9)
100–150	33 (16.3)	8 (32.0)	37 (33.3)
150–200	12 (5.9)	0	3 (2.7)
200–300	8 (3.9)	0	20 (18.0)
			*p* < 0.0001 ^(a)^
<50	76 (37.4)	13 (52.0)	20 (18.0)
			*p* = 0.0002 ^(b)^/*p* = 0.0008 ^(c)^
>150	20 (9.9)	0	23 (20.7)
			*p* = 0.0069 ^(b)^/*p* = 0.0058 ^(c)^

*n*, number of women. ^(a)^ Chi-square test, five degrees of freedom (vs. 2006/07 untreated group); ^(b)^ Fisher’s exact test (vs. 2006/07 untreated group); ^(c)^ Fisher’s exact test (vs. 2018/19 untreated group).

**Table 2:** Urinary iodine concentration (UIC) according to gestational period.

Gestation Trimester	UIC (µg/L)	Year 2006/07 (No Iodine Supplementation)	Year 2018/19 (No Iodine Supplementation)	Year 2018/19 (200 µg/Day Iodine Supplementation)
		*n* (%)	*n* (%)	*n* (%)
First	<50	6 (30.0)	2 (40.0)	1 (16.7)
	50–150	10 (50.0)	3 (60.0)	3 (50.0)
	>150	4 (20.0)	0	2 (33.3)
		(median UIC = 78.6 µg/L)	(median UIC = 116.7 µg/L)	(median UIC = 125.0 µg/L)
Second	<50	16 (30.2)	5 (62.5)	7 (18.9) ^(a)^
	50–150	33 (62.3)	3 (37.5)	21 (56.8)
	>150	4 (7.5)	0	9 (24.3) ^(b)^
		(median UIC = 73.9 µg/L)	(median UIC = 43.8 µg/L)	(median UIC = 109.1 µg/L)
Third	<50	52 (41.9)	6 (50.0)	12 (17.6) ^(c, d)^
	50–150	60 (48.4)	6 (50.0)	44 (64.7)
	>150	12 (9.7)	0	12 (17.6)
		(median UIC = 62.2 µg/L)	(median UIC = 50.0 µg/L)	(median UIC = 101.5 µg/L)

*n*, number of women. ^(a)^ Fisher’s exact test, *p* = 0.0224 (vs. 2018/19 untreated group); ^(b)^ Fisher’s exact test, *p* = 0.0279 (vs. 2006/07 untreated group); ^(c)^ Fisher’s exact test, *p* = 0.0004 (vs. 2006/07 untreated group); ^(d)^ Fisher’s exact test, *p* = 0.0228 (vs. 2018/19 untreated group). Chi-square test for trend across gestational trimesters showed no significant differences in any of the groups.

## Data Availability

The data that support the findings of this study are available from the corresponding author upon reasonable request.
